# Prevalence of polypoidal choroidal vasculopathy in Indian population: Risk factors, clinical and imaging characteristics

**DOI:** 10.1371/journal.pone.0231901

**Published:** 2020-04-28

**Authors:** Meenakshi Kumar, Sangeetha E. Moptom, Parveen Sen, Vikas Khetan, Muna Bhende, Sobha Sivaprasad, Rajiv Raman, on behalf of Sankara Nethralaya Vitreoretinal Study Group (SNVR-Study Group)

**Affiliations:** 1 Shri Bhagwan Mahavir Vitreoretinal Services, Sankara Nethralaya, Chennai, India; 2 Sankara Nethralaya Diabetic Retinopathy project, Chennai, India; 3 NIHR Moorfields Biomedical Research Centre, London, United Kingdom; University Hospitals Cleveland, UNITED STATES

## Abstract

**Aim:**

To assess prevalence, clinical presentation and multimodal imaging characteristics of polypoidal choroidal vasculopathy (PCV) in a hospital-based setting in South India.

**Methods:**

Electronic medical records (EMR) of new patients presenting with suspected clinical signs of wet age-related macular degeneration (AMD) in a tertiary hospital from January to December 2016 were retrospectively analyzed using keywords and filtered for patient who underwent multimodal imaging. Clinical presentations were categorized into predominantly hemorrhagic, exudative or mixed pattern. The imaging features were compared in these clinical groups. The multimodal images were graded by two masked graders and discrepancies between them were settled by a senior arbitrator.

**Results:**

Of the 147 clinically suspicious cases of PCV out of 785 patients with clinical presentation of AMD as recorded in the EMR, 73 (49.7%) patients had a multimodal imaging diagnosis of PCV. There was no difference in the demography, distribution of polyps, ICGA and OCT characteristics in eyes presenting with hemorrhagic, exudative or mixed clinical features.

**Conclusion:**

Approximately half of South Asian patients presenting with clinical features of neovascular AMD harbor PCV irrespective of their clinical presentation and so we recommend that multimodal imaging is done in all cases of suspicious neovascular AMD in Indian population.

## Introduction

In contrast to epidemiological studies on age related macular degeneration (AMD), there are very few studies on population-based data on polypoidal choroidal vasculopathy (PCV) due to the inherent difficulty of diagnosing the disease from fundus photographs alone [1]. Thus, the estimates of PCV prevalence can only be accurately derived from hospital based cross-sectional studies. These studies have shown a prevalence of 4% [2] to 54% [3] depending on the inclusion criteria, imaging modality and ethnic origin of the population. The prevalence of PCV is known to be higher in Afro-Caribbean cohort and Eastern Asia population and low in the white race [4].

Although PCV has characteristic optical coherence tomography (OCT) features, indocyanine green angiography (ICGA) remains the definitive diagnostic imaging modality for PCV [5]. We have previously reported the prevalence of early or intermediate AMD to be ~ 21% in the rural Indian population and ~ 16% in the urban Indian population aged 60 years or more based on color fundus photographs. Likewise, the prevalence of neovascular AMD is ~ 2% in the same rural and urban Indian populations [6]. However, ICGA was not done in any population-based studies from India and so the prevalence of PCV in India is unclear. The aim of the study was to elucidate the prevalence, demography, risk factors and imaging characteristics of PCV in a hospital-based South Indian population presenting with characteristic features of neovascular AMD.

## Methods

All patients aged 40 years or above who presented with presumed neovascular AMD to the Vitreoretinal services of a tertiary eye care center in South India from January to December 2016 were reviewed from the electronic medical records (EMR). Institutional review board approval (Ethics Committee, Vision Research Foundation) was obtained for this retrospective study. It is the hospital protocol to obtain informed consent form before any procedure including invasive procedure such as ICGA and FFA. The informed consent includes the information on how data will be used for scientific research and will be anonymized. The EMR was searched using the following keywords that defined the presenting diagnosis: choroidal neovascular membrane, wet age-related macular degeneration, hemorrhagic pigment endothelial detachment, polyps and ICD coding for polypoidal choroidal vasculopathy. All these entities represent the spectrum of serosanguinous maculopathy. Those with clinical suspicion of PCV, that is presence of subretinal exudation, subretinal hemorrhage, presence of pigment epithelial detachment, serous neurosensory detachment, peripapillary location, multiple lesions and visible polyps, underwent multimodal imaging. Multimodal imaging included color fundus photograph (Visupac, Version 4.4.3, Carl Zeiss Meditec.), fundus fluorescein angiography (FFA), ICGA (HRA system; Heidelberg Engineering, Heidelberg, Germany and visupac, Version 4.4.3, Carl Zeiss Meditec) and OCT (Cirrus OCT-Carl Zeiss Meditec, Dublin, CA, USA, Spectralis OCT- HRA system; Heidelberg Engineering, Heidelberg, Germany, Swept Source OCT– 3D Optical Coherence tomography: DRI OCT-1 Atlantis, Topcon 3D OCT 2000-Toyko,Japan.). The extracted images were deidentified.

### Clinical presentations of PCV

The clinical presentations were graded as (1) predominantly hemorrhagic pattern defined as presence of hemorrhagic pigment epithelial detachment (PED), submacular hemorrhage and multiple layers of hemorrhage, or (2) exudative pattern defined as the presence of exudates with serous or serosanguinous PED and/or subretinal fluid or (3) mixed lesions defined as the presence of both hemorrhagic and exudative features.

### Multimodal imaging diagnosis of PCV

The diagnosis of PCV on colour photographs FFA and ICGA were confirmed based on the EVEREST criteria [7] that included the presence of early sub-retinal focal hyperfluorescence on ICGA (within the first 6 min), and at least one of the following criteria: (1) nodular appearance of the polyp(s) on stereoscopic examination, (2) hypofluorescent halo around the nodule(s), (3) presence of a branching vascular network (BVN), (4) pulsation of the polyp(s) on dynamic ICGA, (5) orange sub-retinal nodules on color fundus photography that corresponded to the ICGA nodules, or (6) massive sub-macular hemorrhage (≥4 disc areas in size). Definitive polyp was defined as the presence of aneurysmal dilation clearly demarked in ICGA as minute hyperfluorescence. When the appearance of minute hyperfluorescence or aneurysmal dilation was masked due to hemorrhage or media haze and there was diffuse hyperfluorescence suggestive of an underlying polyp, the term suspicious polyp was used. Choroidal neovascularization (CNVM) due to AMD was diagnosed if the lesion showed presence of subretinal hemorrhage, subretinal fluid and retinal pigment epithelium(RPE) alterations with occult, ill-defined choroidal neovascularization lesion on FFA and early hyperfluorescence spots or plaque like hyperfluorescence in ICGA along with the absence of polyp and abnormal branching vascular network. Likewise, those who had features of different stages of retinal angiomatous proliferans (RAP) as described by Yannuzzi et al [8] were also excluded.

### Specific lesion characteristics of PCV

**Fig 1:**
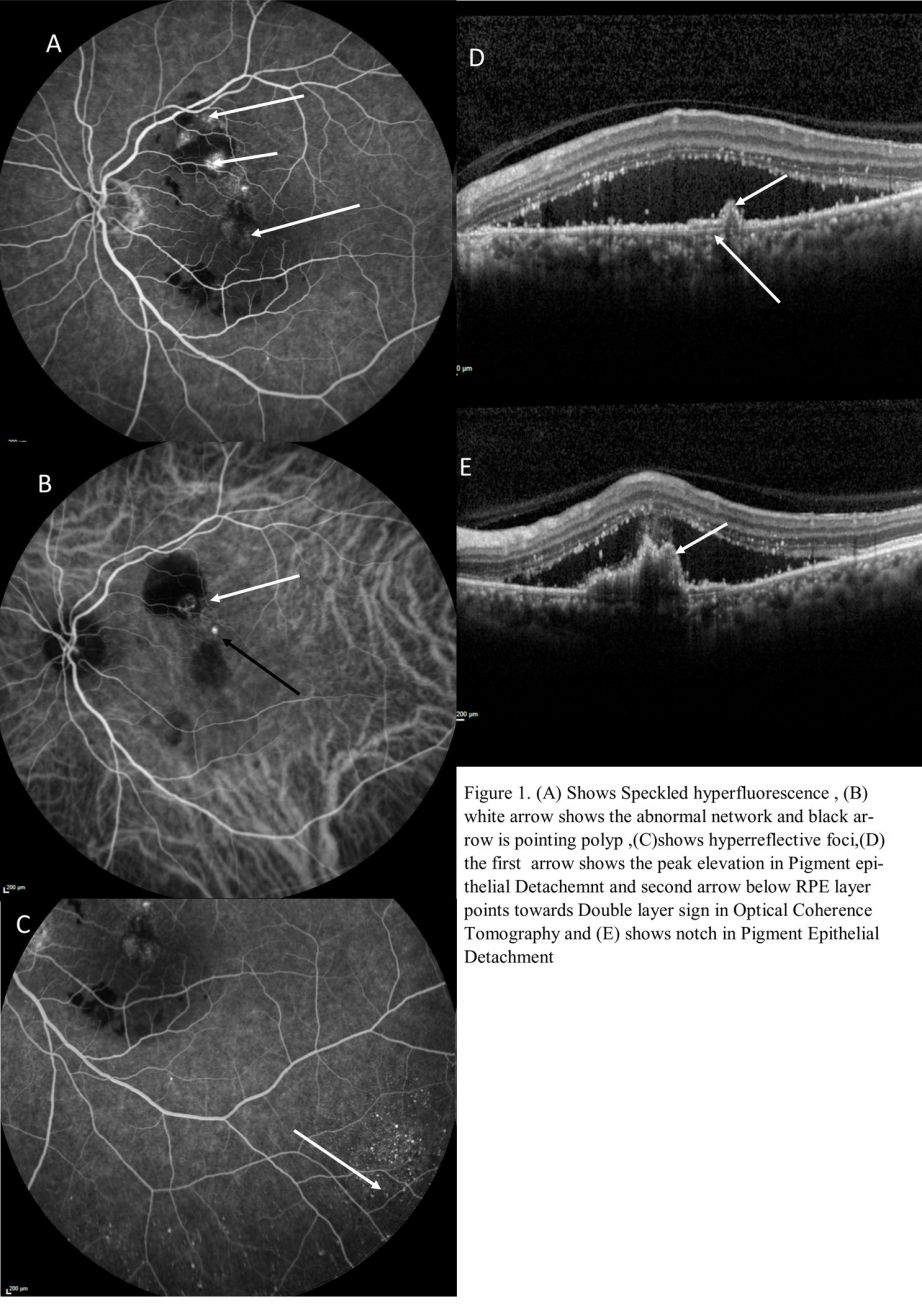
Definitions used in the classification of features.

OCT and ICGA features of lesion characteristics were further defined as described as in the report by Anantharaman et al [9] and is shown in Fig 1. BVN (Branching vascular network) was defined on early phase of the ICGA (first 1 min) as a distinct network of vessels within the choroid termed as branching vascular network. Polyp was defined as small hyperfluorescent spots, isolated or in clusters that become visible soon after the PCV network is discernible on the ICGA. Early nodular hyperfluorescence arising from choroidal circulation noted within the first 6 min of dye injection were termed polyp. The polyps also leak slowly as the surrounding hitherto hypofluorescent area becomes increasingly hyper-fluorescence. Choroidal hyperpermeability was defined on mid phase ICGA as multiple and patchy choroidal hyperfluorescence.

#### Speckled hyperfluorescence

Mid phase multiple and patchy choroidal hyperfluorescence, indicating choroidal hyperpermeability.

Late-onset focal hyperfluorescent lesion with clear margin apparent with rosette pattern after at least 6 minutes phase of ICGA was termed as hyperfluorescence foci. OCT characteristics included peak-like PED defined as a sharp peak like elevation seen in PED with underlying moderate reflectivity. A tomographic notch was defined as a V-shaped dip between two PED and a double layer sign (DLS) was defined as a separation of RPE layer from underlying Bruch’s membrane containing neovascular complex.

### Accuracy of grading

The extracted images were deidentified and were assessed by 2 senior retina specialists (with more than 10 years retinal experience) independently in a masked manner. An adjudicated grade by a third senior retina specialist was considered as a final diagnosis in cases where the diagnosis differed between the two retina specialists.

### Statistical analysis

Statistical analysis was performed using statistical software (SPSS for windows, Version 20, SPSS science, IBM, Chicago, Illinois, USA). The results were expressed in frequencies for categorical data and percentage for continuous data. Chi square test was done to compare proportions in PCV and CNVM group. RAPs were excluded from comparative analysis due to the small numbers. Univariate logistic regression analysis was performed to assess risk factors for PCV. Kappa statistics was done to report intergrader agreement.

## Results

**Fig 2:**
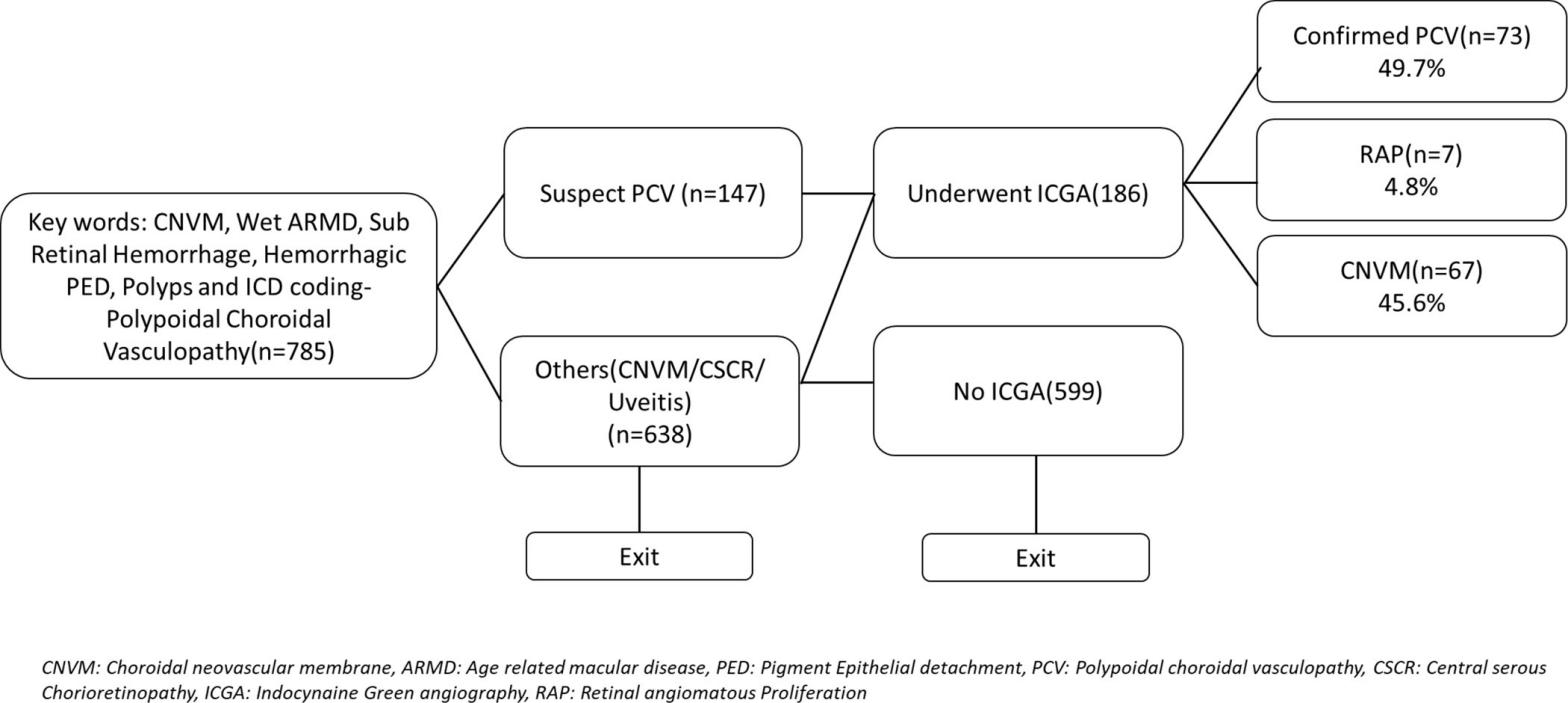
Flow of patient search and grading process.

Of the 785 records that matched with the keywords searched, 147 patients underwent multimodal imaging due to clinical suspicion of PCV. The proportions of patients with PCV, CNVM and RAP based on clinical presentations are shown in Fig 2. The prevalence of PCV among serosanguinous maculopathy was found to be 49.7% (Fig 2). The overall prevalence of PCV was 9.29%. The kappa agreement between the graders was k = 0.632 (p = 0.001).

Table 1 shows the demographic, systemic and ocular features of PCV. The prevalence of PCV increased with increasing age with a maximum (38.4%) in 70–80 years age group. PCV showed a unilateral preponderance (82.2%) and the majority were located in the macula (64.4%). The clinical presentation included hemorrhagic (52.1%), followed by exudative (34.25%) and mixed (13.70%). History of hypertension, ischemic heart disease were less prevalent in PCV.

**Table 1:** Prevalence of PCV in different subgroups.

Variable	n	Prevalence	95%CI	p
**Age (years)**				
40–50	8	10.95%	(5.66–20.66)	**0.00**
50–60	11	15.06%	(8.63–25.00)	
60–70	28	38.35%	(28.05–49.83)	
70 and above	26	36.99%	(26.82–48.45)	
**Gender**				
Male	38	52.05%	(40.78–63.12)	0.725
Female	35	47.95%	(36.87–59.21)	
**Laterality**				
Unilateral	60	82.20%	(71.88–89.29)	**0.00**
Bilateral	13	17.80%	(10.71–28.12)	
**Location**				
Macular	47	64.38%	(52.93–74.41)	**0.00**
Extra-macular	11	15.07%	(8.63–25.00)	
Peripapillary	6	8.22%	(3.82–16.79)	
Mixed	9	12.33%	(6.62–21.80)	
PCV* **Type**				
Exudative	25	34.25%	(24.39–45.67)	**0.00**
Hemorrhagic	38	52.10%	(40.78–63.12)	
Mixed	10	13.70%	(7.61–23.41)	
**Presence of Systemic disease**				
**H/o**^†^ **Diabetic mellitus**				
Present	30	58.90%	(47.44–69.46)	0.13
Absent	43	41.10%	(30.53–52.55)	
**H/o**^†^ **Hypertension**				
Present	37	50.68%	(39.47–61.83)	0.907
Absent	36	49.32%	(38.17–60.53)	
**H/o**^†^ **Ischemic heart disease**				
Present	9	12.33%	(6.62–21.80)	**0.00**
Absent	64	87.67%	(78.20–93.38)	
**H/o**^†^ **Hypercholesterolemia**				
Present	9	9.59%	(4.72–18.50)	**0.00**
Absent	64	87.67	(78.20–93.38)	

*PCV: Polypoidal Choroidal vasculopathy,

^†^H/o: History.

Table 2 shows the univariate and multivariate analysis of risk factors for PCV. No statistical significance was achieved.

**Table 2:** Univariate and multivariate analysis for risk factors for PCV.

Variable	Univariate		Multivariate	
	*OR(95% CI)*	*P*	*OR(95% CI)*	*p*
Gender	1.59(0.810–3.038)	0.18	1.59(0.812–3.11)	0.174
H/o Diabetic mellites	1.51(0.769–2.991)	0.229	1.44(0.695–2.995)	0.325
H/o Hypertension	1.31(0.687–2.52)	0.408	1.198(0.608–2.364)	0.601
H/o Ischemic heart disease	1.00(0.873–2.683)	1	0.899(0.320–2.528)	0.84
H/o Hypercholesterolemia	1.83(0.512–6.54)	0.353	1.44(0.390–5.634)	0.598

PCV: Polypoidal choroidovasculopathy, H/o: History, OR: Odds ratio.

**Fig 3:**
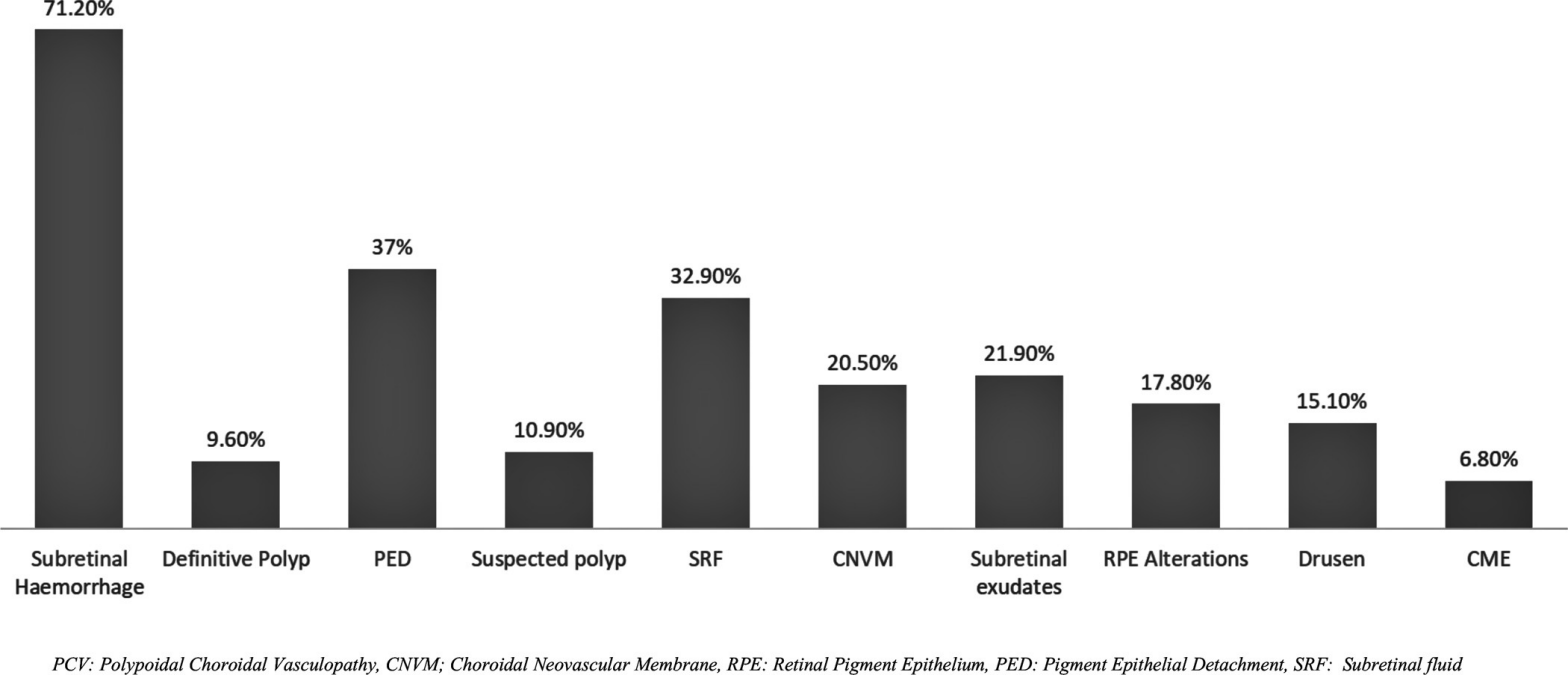
The proportions of patients with PCV, CNVM and RAP in each clinical category.

52.10% of PCV patients presented with hemorrhagic pattern, 34.25% showed exudative and 13.70% were of mixed subtype. Fig 3 shows the frequency of clinical findings present on fundus photographs. Most common feature was the presence of subretinal hemorrhage (71.2%) followed by PED (37%). Definitive clinically polyp was visible in 9.6% cases and suspicious polyps were present in 10.9% cases. No pulsation of polyp was observed in these cases.

Table 3 shows the FFA and ICGA features of patients with PCV. The presence of BVN was noted in 58% of the PCV patients and it was maximum in hemorrhagic type of PCV. However statistically significant differences in the presence of BVN were not observed in the three types of PCV. Polyps were single in 34.3% cases, multiple in 39.7% and in cluster in17.8% cases. The occurrence of speckled hyperfluorescence was seen in 54.8% cases, however it was not statistically significant across the groups. The presence of hyperfluorescent foci was noted only in 17.8% of the PCV cases.

**Table 3:** Fundus fluorescein and Indocynanine green angiogram features in patients with PCV.

Feature	Exudative (N = 25)	Hemorrhagic (N = 38)	Mixed (N = 10)	p	Overall (N = 73)
	n (%)	n (%)	n (%)		n (%)
BVN*					
Present	13 (52%)	22 (58%)	7 (70%)	0.62	42 (58%)
Absent	12 (48%)	16 (42%)	3 (30%)		31 (42%)
**Polyp**					
None	2 (8%)	3 (7.9%)	1 (10%)	0.09	6 (8.2%)
Single	9 (36%)	13 (34.2%)	3 (30%)		25 (34.3%)
Multiple	9 (36%)	17 (44.7%)	3 (30%)		29 (39.7%)
Cluster	5 (20%)	5 (13.2%)	3 (30%)		13 (17.8%)
**Speckled hyperfluorescence**					
Present	11 (44%)	21 (55.3%)	8 (80%)	0.15	40 (54.8%)
Absent	14 (56%)	17 (44.7%)	2 (20%)		33 (45.2%)
**Hyperfluorescence foci**					
Present	5 (20%)	7 (18.4%)	1 (10%)	0.78	13 (17.8%)
Absent	20 (80%)	31 (81.6%)	9 (90%)		60 (82.2%)

*BVN: Branching vascular network.

Table 4 shows the OCT characteristics of PCV across the three subtypes. Peaked PED was seen in 67.1%, tomographic notch in 68.5%- and double-layer sign in 61.6% of the cases. There were no statistically significant differences in the distribution of these features in the clinical subtypes of PCV.

**Table 4:** OCT features in the patients with PCV.

Feature	Exudative (N = 25)	Hemorrhagic (N = 38)	Mixed (N = 10)	p	Overall (N = 73)
	n (%)	n (%)	n (%)		n (%)
PED*					
None	0 (0%)	1 (2.6%)	0 (0%)		1 (1.4%)
Fibrovascular PED*	8 (32%)	5 (13.2%)	3 (30%)	0.093	16 (21.9%)
Hemorrhagic PED*	2 (8%)	14 (36.8%)	1 (10%)		17 (23.3%
Serosanguinous PED*	15 (60%)	18 (47.4%)	6 (60%)		39 (53.4%)
**SRF**^†^					
Present	20 (80%)	35 (92.1%)	9 (90%)		64 (87.7%)
Absent	5 (20%)	3 (7.9%)	1 (10%)	0.35	9 (12.3%)
**SRH**^‡^					
Present	10 (40%)	29 (76.3%)	8 (80%)	**0.007**	47 (6.4%)
Absent	15 (60%)	9 (23.7%)	2 (20%)		26 (93.6%)
Peaked PED*					
Present	16(64%)	27(71.05%)	6(60%)	0.735	49 (67.1)%
Absent	9(36%)	11(28.95%)	4(40%)		24 (32.8%)
**Tomographic notch**					
Present	18(72.5)	26(68.4%)	6(60%)	0.785	50 (68.5%)
Absent	7(28%)	12(31.6%)	4(40%)		23 (31.5%)
**Double Layer sign**					
Present	13(52%)	24(63.1%)	8(80%)		45 (61.6%)
Absent	12(48%)	14(36.8%)	2(20%)	0.294	28 (38.4%)

*PED: Pigment epithelial detachment,

^†^SRF: Subretinal fluid,

^‡^SRH: Subretinal hemorrhage,

## Discussion

We report the prevalence of clinical and imaging characteristics of PCV in an Indian population seen within a tertiary center in South India. The prevalence of PCV among patients with serosanguinous maculopathy was found to be 49.7%, overall prevalence being 9.29% and is comparable to other clinic-based population studies conducted in Asia and Europe population.

Table 5 shows the summary of hospital and population- based studies across different population. Similar to our study, majority of the studies are hospital- based studies. The prevalence of PCV is lower in studies from US [5] and Europe [2,10,11] compared to studies from Asia. [4,12,13–15,16] It is unknown why such ethnic variation exists in the epidemiology of PCV. Although a genetic predisposition may explain racial differences [17], it is unclear why the deeper choroidal vessels are more affected in Asian and Afrocarribean populations compared to the choriocapillaris in the white races. Further studies on differences in scleral resistance, vortex vein and choroidal vasculature may provide clues to explain these differences.

**Table 5:** Comparing the prevalence of PCV across various studies.

Author	Year	Country	Mean age (years)	Diagnosis tools used	Study Population	N	Unilateral%	Prevalence
Yannuzzi et al [5]	1999	USA	74.4	ICGA* features	Hospital based	167	46.2	7.80%
Lafaut et al [2]	2000	Belgium	NA	FP^†^, FFA** and ICGA*	Hospital based	374 eyes	39	4.00%
Scassellati–sforzolini et al [10]	2001	Italy	70.2	FFA** and ICGA* features	Hospital based	194	78.9	9.80%
Kwok et al [18]	2002	China	65.1	ICGA* features	Hospital based	19	84.2	9.30%
Sho et al [12]	2003	Japan	68.4	FP^†^ and ICGA*	Hospital based	418	90	23.92%
Ladas et al [19]	2004	Greece	72.5	ICGA* features	Hospital based	268	45.5	8.20%
Wen et al [13]	2004	China	68.3	FP^†^,FFA** and ICGA	Hospital based	166	86.5	22.30%
Maruko et al [3]	2007	Japan	NA	ICGA* features	Hospital based	289	82.9	54.70%
Liu et al [14]	2007	China	65.4	FP^†^,FFA** and ICGA*	Hospital based	155	76.3	24.50%
Beyon et al [15]	2008	Korea	64.6	FP^†^,FFA** and ICGA*	Hospital based	79	75.9	24.60%
Song et al [20]	2009	Korea	57.2	FP^†^,FFA** and ICGA*	Population based	10890	90	22.20%
Li et al [21]	2014	China	NA	OCT^‡^	Population based	3468	94.1	0.30%
Cackett et al [16]	2010	China	68.3	ICGA* features	Hospital based	123	87.8	55.6%
Bhoomibunchoo et al [4]	2017	Thailand	59.5	FP^†^ and ICGA*	Hospital based	140	93	77.50%
Yadav et al [11]	2017	UK	75.4	ICGA* features	Hospital based	492	NA	9.14%
Our study	2018	India	65.3	FP^†^,FFA**,OCT^‡^ and ICGA*	Hospital based	147	82.2	9.29%

*ICGA:Indocyanine green angiogram,

^†^FP; fundus photo,

^‡^OCT: Optical coherence tomography,

**FFA: Fundus fluorescein angiogram.

We found no difference in gender predilection between PCV and CNVM, similar to that reported by Yannuzzi et al [5] and Scassellati–sforzolini et al [10]. Previous reports indicate that PCV is more prevalent in men in Asian populations (22–37% female), but the opposite is observed in Caucasians (52–65% female). Differences in disease characterization and study design may explain our observation.

Surprisingly, we did not find a significant association of hypertension in PCV compared to CNVM group. The first literature is black ladies with hypertension [22].However, subsequent literature is conflicting. Ahuja et al [23] reported that 2.90% of their series had diabetes and 23.5% had a history of hypertension. Ladas et al [19] reported, 36.4% patients had a history of hypertension. Byeon et al [15] and Sho et al [12] in their series found that 31.65% and 36% had history of hypertension and 12.66% and 11% had a history of diabetes respectively. In our series 37 (50.68%) patients with PCV were hypertensive and 41.09% had history of diabetes. Given that hypertension is highly prevalent in this age group in the Asian population, an association of hypertension and PCV cannot be elucidated from small clinic-based studies.

Our study confirmed that macular polyps are more common in Asian population. PCVs were initially thought to occur in the peripapillary location. Subsequently, PCV lesions have been described in macular and mid periphery with ethnic variations. In our study, majority of polyps were macular (64.4%), followed by extra macular group 15.1%. Peripapillary polyps were found in only 8.2% of the eyes. Umaya et al [24] observed 94% polyps were macular and 9% were peripapillary in their study on 14 eyes. Anantharaman et al [9] also reported 45% being subfoveal 18% juxta foveal polyps and 18% peripapillary polyps in their study on 45 eyes in Indian patients. In contrast, studies from Europeans showed an equal distribution of macular and peripapillary location [25].

Our study showed that the prevalence increased with increasing age; majority being unilateral and macular. Hemorrhagic PCVs were slightly more common than the exudative subtypes. Sho et al [12] in his series reported 59% had exudative pattern and 30% had hemorrhagic pattern. Byeon et al [15] also reported higher rates of exudative pattern in 52% and hemorrhagic pattern in 34.7% of cases. Similar to our study, Bhoomibunchoo et al [4] reported hemorrhagic pattern to be 88.79% and exudative pattern in 11.21%. Uyama et al [24] and Ahuja et al [23] also reported higher percentage of hemorrhagic pattern which correlates with our study. We observed that the ICGA and OCT features of PCV do not statistically vary between the three main clinical presentations of PCV.

**Fig 4:**
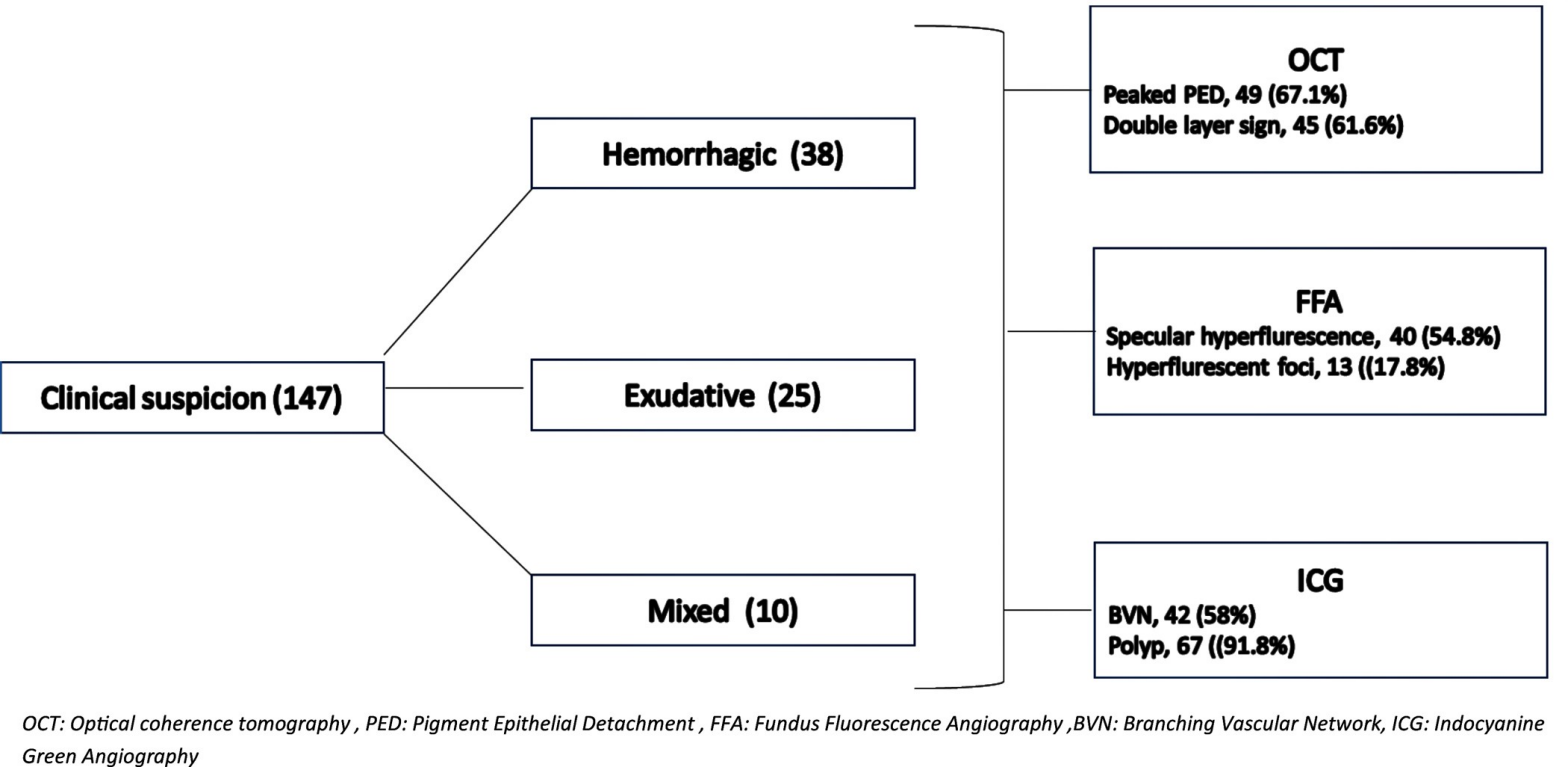
Multiple modalities role in PCV diagnosis.

Fig 4 shows that despite multiple modalities like FFA and OCT, ICG was the most helpful in diagnosis of PCV. The polyps were seen in 91.8% cases. However, features picked up on multimodal imaging collaborates in the diagnosis of PCV.

The merit of the present study is that it is the first study to examine the prevalence, risk factors and imaging characteristics of PCV in an Indian population from a tertiary center. However, our study also has several limitations. First, we may have introduced study sample selection bias because the study population was obtained from the EMR and only patients with multimodal imaging were selected. Our study results indicate that PCV may be present irrespective of the clinical presentation, suggesting that our study may have under-reported the prevalence of PCV. However, a prospective, multimodal imaging in a population-based prevalence study of PCV in an Indian population is not a feasible option.

Second, the possibility of potential bias in ascertaining history-related variables from EMR records should also be kept in mind; this was true particularly with regard to diabetes, hypertension and hypercholesteremia.

In conclusion, in the present study, we found that PCV is present in approximately half our study cohort and the prevalence, OCT and ICGA characteristics of PCV are similar irrespective of the clinical presentation. Therefore, multimodal imaging is recommended for all patients presenting with signs and symptoms of neovascular AMD.
